# Overlooked and underpowered: a meta-research addressing sample size in radiomics prediction models for binary outcomes

**DOI:** 10.1007/s00330-024-11331-0

**Published:** 2025-01-09

**Authors:** Jingyu Zhong, Xianwei Liu, Junjie Lu, Jiarui Yang, Guangcheng Zhang, Shiqi Mao, Haoda Chen, Qian Yin, Qingqing Cen, Run Jiang, Yang Song, Minda Lu, Jingshen Chu, Yue Xing, Yangfan Hu, Defang Ding, Xiang Ge, Huan Zhang, Weiwu Yao

**Affiliations:** 1 Laboratory of Key Technology and Materials in Minimally Invasive Spine Surgery, Tongren Hospital, Shanghai Jiao Tong University School of Medicine, Shanghai, China; 2 Center for Spinal Minimally Invasive Research, Shanghai Jiao Tong University, Shanghai, China; 3 Department of Imaging, Tongren Hospital, Shanghai Jiao Tong University School of Medicine, Shanghai, China; 4 Department of Epidemiology and Population Health, Stanford University School of Medicine, Stanford, CA USA; 5 Department of Biomedical Engineering, Boston University, Boston, MA USA; 6 Department of Orthopedics, Shanghai Sixth People’s Hospital, Shanghai Jiao Tong University School of Medicine, Shanghai, China; 7 Department of Medical Oncology, Shanghai Pulmonary Hospital, Tongji University School of Medicine, Shanghai, China; 8 Department of General Surgery, Pancreatic Disease Center, Ruijin Hospital, Shanghai Jiao Tong University School of Medicine, Shanghai, China; 9 Department of Pathology, Shanghai Sixth People’s Hospital, Shanghai Jiao Tong University School of Medicine, Shanghai, China; 10 Department of Dermatology, Shanghai Ninth People’s Hospital, Shanghai Jiao Tong University School of Medicine, Shanghai, China; 11 Department of Pharmacovigilance, SciClone Pharmaceuticals (Holdings) Ltd., Shanghai, China; 12 MR Scientific Marketing, Siemens Healthineers Ltd., Shanghai, China; 13 MR Application, Siemens Healthineers Ltd., Shanghai, China; 14 Editorial Office of Journal of Diagnostics Concepts & Practice, Department of Science and Technology Development, Ruijin Hospital, Shanghai Jiao Tong University School of Medicine, Shanghai, China; 15 Department of Radiology, Ruijin Hospital, Shanghai Jiao Tong University School of Medicine, Shanghai, China

**Keywords:** Sample size, Methodology, Prediction model, Radiomics

## Abstract

**Objectives:**

To investigate how studies determine the sample size when developing radiomics prediction models for binary outcomes, and whether the sample size meets the estimates obtained by using established criteria.

**Methods:**

We identified radiomics studies that were published from 01 January 2023 to 31 December 2023 in seven leading peer-reviewed radiological journals. We reviewed the sample size justification methods, and actual sample size used. We calculated and compared the actual sample size used to the estimates obtained by using three established criteria proposed by Riley et al. We investigated which characteristics factors were associated with the sufficient sample size that meets the estimates obtained by using established criteria proposed by Riley et al.

**Results:**

We included 116 studies. Eleven out of one hundred sixteen studies justified the sample size, in which 6/11 performed a priori sample size calculation. The median (first and third quartile, Q1, Q3) of the total sample size is 223 (130, 463), and those of sample size for training are 150 (90, 288). The median (Q1, Q3) difference between total sample size and minimum sample size according to established criteria are −100 (−216, 183), and those differences between total sample size and a more restrictive approach based on established criteria are −268 (−427, −157). The presence of external testing and the specialty of the topic were associated with sufficient sample size.

**Conclusion:**

Radiomics studies are often designed without sample size justification, whose sample size may be too small to avoid overfitting. Sample size justification is encouraged when developing a radiomics model.

**Key Points:**

***Question*** *Sample size justification is critical to help minimize overfitting in developing a radiomics model, but is overlooked and underpowered in radiomics research*.

***Findings*** *Few of the radiomics models justified, calculated, or reported their sample size, and most of them did not meet the recent formal sample size criteria*.

***Clinical relevance*** *Radiomics models are often designed without sample size justification. Consequently, many models are too small to avoid overfitting. It should be encouraged to justify, perform, and report the considerations on sample size when developing radiomics models*.

## Introduction

Radiomics is a method that extracts high-dimensional minable features from medical images to reveal the complex patterns and biological basis of a specific disease that is undetectable by the naked eye [1, 2]. These radiomics features can be used as candidate predictor parameters for building diagnostic or prognostic models that have the potential to aid clinical decision-making [3–7]. In contrast to the increasing number of research papers that publish various models [8, 9], the majority of the radiomics models failed to be translated into clinical practice [10, 11]. The gap between academic radiomics research and clinically practicable tools may arise from a sometimes lower-than-desired robustness of reported results over a wider variety of samples [12–17]. One of the important methodological items that should be considered in developing a prediction model is the sample size [18–22]. It has been pointed out that the pitfall of developing a radiomics model with a small sample size can lead to overfitting [6, 23–26].

Unfortunately, methodological research in sample size determination is scarce in the medical imaging field [27, 28]. There is currently no well-adopted method for determining the sample size in radiomics research [29–33]. One may consider that the limitation of a small sample size can be overcome by using penalization and shrinkage methods, such as least absolute shrinkage and selection operator regression, which is usually employed in radiomics research. However, the shrinkage parameters are uncertain when they are estimated with a small sample size [15, 34]. A rule of thumb of events per variable (EPV) has been widely used to guide the calculation and justification of the sample size [28, 35]. It is easy to use and might be a pragmatic approach to identifying a reasonable target size in some cases. Nevertheless, it has been widely cautioned against, since the evidence base for the rule of thumb is simulation studies that investigate the performance of estimating covariate-outcome relationships [36]. Additional sample size determination processes including model-based considerations or the learning curve-fitting approach [37–39] may be appropriate in other cases, but they cannot be easily applied by general researchers.

To prompt the implementation of sample size calculation, a series of papers have been published to guide the researchers in calculating the minimum sample size for their prediction model research [40–46]. The calculation according to Reily et al can be easily conducted as it has been provided as a code package for use. This approach determines the minimum sample size to satisfy three criteria [41, 43, 45]: (1) small overfitting defined by an expected shrinkage of predictor effects by 10% or less, (2) small absolute difference of 0.05 in the model’s apparent and adjusted Nagelkerke’s *R*^2^ value, and (3) precise estimation (within ± 0.05) of the average outcome risk in the population. However, it is unknown whether this approach is adhered to in radiomics research, or if other approaches are prevalent, such as the rule of EPV ≥ 10.

In this study, we aimed to (1) evaluate if and how sample size was justified in recently published radiomics prediction models for binary outcomes, and (2) calculate the minimum required sample size required for each radiomics model and compare it with the actual available sample size.

## Materials and methods

### Study design and reporting

This is a meta-research study that focuses on the sample size determination of the published literature on radiomics models [47, 48]. Institutional ethical approval or written informed consent is not required because of the nature of this study. We have drafted a study protocol in advance (Supplementary Note S1), and registered and uploaded materials for this study on the open science framework (https://osf.io/pbukc/).

### Literature search and study selection

Our study included primary studies that developed a radiomics prediction model for a binary, patient-related health outcome published online from 01 January 2023 to 31 December 2023 in seven leading peer-reviewed radiology journals (*European Radiology*, *Insights into Imaging, European Radiology Experimental*, *Radiology*, *Radiology: Artificial Intelligence*, *Radiology: Cardiothoracic Imaging*, and *Radiology: Imaging Cancer*) (Supplementary Note S2). These journals were selected, because they are owned by two large radiological societies, and are listed in the Science Citation Index Expanded, or Emerging Science Citation Index, in Radiology, Nuclear Medicine and Medical Imaging Category, 2023 Journal Citation Reports (https://jcr.clarivate.com/jcr/home). We searched the PubMed database (www.pubmed.ncbi.nlm.nih.gov) for potentially eligible studies using the terms “radiomics” and “radiomic”. We used restrictions on publication time, journals, and publication types to improve the accuracy of the search. The studies were excluded if they were: (1) duplicates; (2) not primary study; (3) study training models using only deep learning methods; (4) studies predicting time-to-event or continuous outcomes; (5) model validation only study or study not aimed to develop prediction models; and (6) study with insufficient data or full-text not available.

Two independent reviewers (J.Z. and X.L.) ran the search strategy on 01 January 2024. The same two reviewers screened the title and abstracts of the records after the exclusion of duplications. The potentially eligible studies were retrieved for full-text and supplementary materials for inclusion. The disagreements were resolved by discussion or by consultation to review the group if necessary. Two independent reviewers (Y.X. and Y.H.) later manually searched the homepages of these seven radiological journals to identify additional potentially eligible studies on 31 March 2024.

### Data extraction and management

Our review group developed a standardized data extraction sheet (Supplementary Note S3) for general information, model development methodology, and model metrics for sample size calculation [46]. The sample size for training, and testing were defined as the sample used to train the model, and the sample used to test the model. The sample size for internal testing, and external testing was defined as the sample used to test the model from the same source of sample size for training, and the sample used to test the model from a geographically different source of sample size for training [15–17].

One reviewer (J.Z.) extracted the data from all included studies using the standardized data extraction sheet, and the results were double-checked by another reviewer (X.L., Y.X., Y.H., D.D, or X.G.). Disagreements were discussed or adjudicated by a review group if necessary.

### Data analysis

The data analysis was carried out by one reviewer (J.Z.) under the supervision of a methodologist (J.L.) using R language (version 4.2.1; https://www.r-project.org/) within RStudio (version 1.3.1093; https://posit.co/) (Supplementary Note S4). The calculation of the minimum sample size was conducted using the *pmsampsize* package (version 1.1.3; https://cran.r-project.org/web/packages/pmsampsize/index.html) [49]. The study characteristics were summarized by frequency and percentages. The sample sizes were presented as median (first and third quartile, Q1 and Q3).

The events per predictor parameter (EPP) was calculated as EPP = the number of events/the number of predictor parameters [50]. We considered the number of predictor parameters in the final radiomics model for the calculation, i.e., the number of radiomics and non-radiomics features that were selected to build the final model. As hundreds of radiomics features would be extracted from the images during the radiomics workflow [29–33], it is nonsense to use the number of extracted features as the candidate predictor parameters for calculation. However, it would be helpful to *post hoc* check whether the sample size for developing the radiomics model is enough to allow the number of features selected, or to a priori calculate how many features the sample size can support.

The sample sizes were calculated using the Riley et al formulae according to three criteria, respectively [41, 43, 45]. The minimum sample size that meets all three criteria needed to be equal to or larger than the largest of the three calculated sample sizes. The calculation was based on the sample size, the number of events, the number of predictor parameters in the model, and the Cox–Snell *R*-square value or the c-statistic [46]. Criterion 3 was separately assessed because this criterion can be considered the absolute lowest sample size that could be accepted when developing a prediction model [50]. We later calculated the difference between the total sample size and minimum required sample size according to Riley et al criterion 3, and the difference between sample size for training and minimum required sample size according to Riley et al all three criteria, respectively [41, 43, 45]. The former was a relatively weak requirement that was easier to achieve in research practice, while the latter was a relatively strict requirement that was a more ideal research approach.

The study would be labeled as sufficient for a sample size that meets the relatively weak and strict requirements, respectively. The characteristics factors including journal, study design, model testing method, imaging modality, specialty of a topic, intended use, presence of calibration metrics, presence of sample size justification, presence of sample size discussion, and application of reporting guidelines, were evaluated to determine whether they were associated with the sufficient sample size that meets the estimates obtained by using established criteria. They were first tested using univariate logistic regression to tell whether they were associated with a sufficient sample size, with an alpha level of 0.10. Multiple logistic regression analysis was used to estimate the adjusted odds ratio (OR) and 95% confidential interval (CI) of whether factors were associated with sufficient sample size, with an alpha level of 0.05.

## Results

### Literature search

**Fig. 1:**
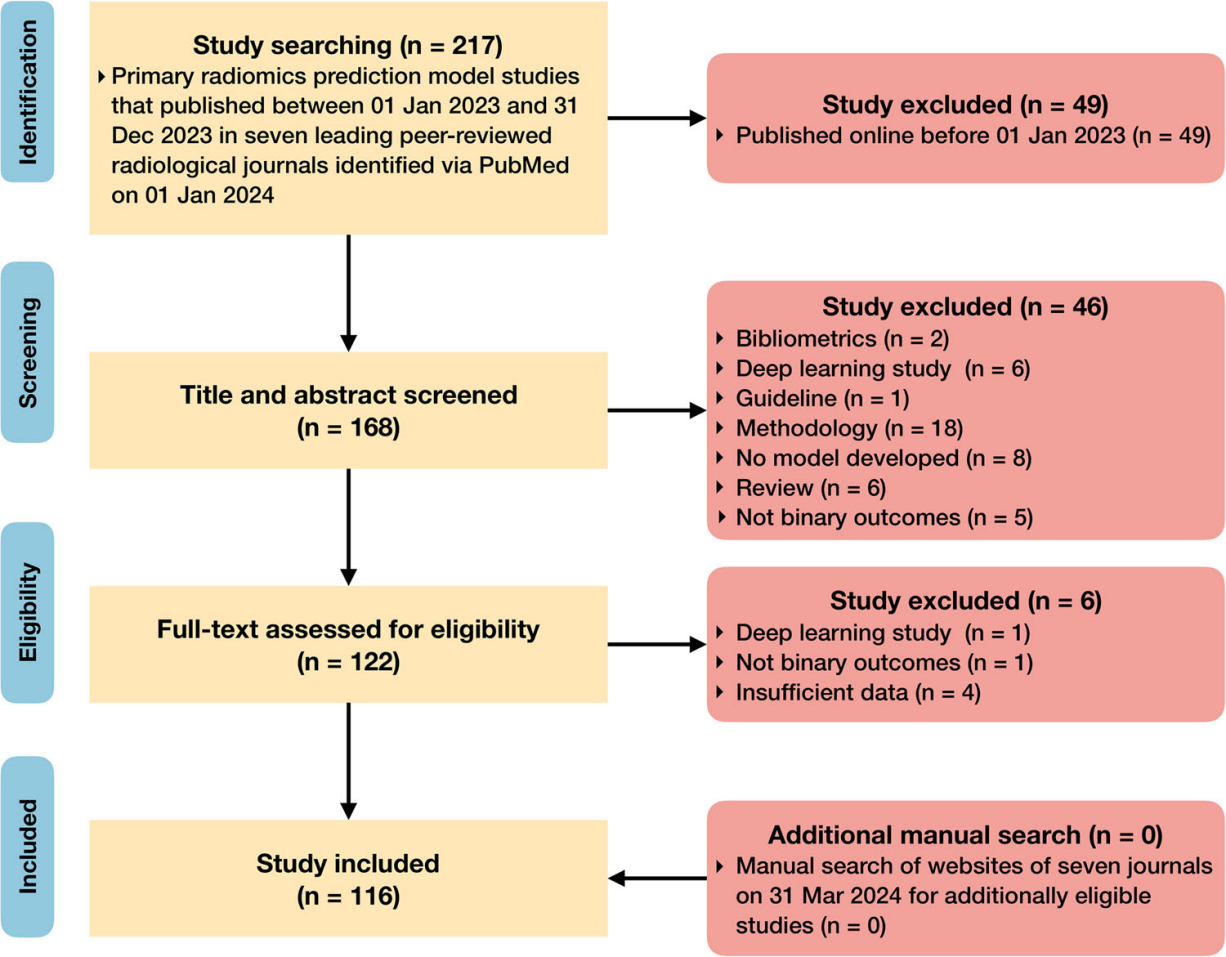
Flow diagram of study inclusion

Our primary literature search yielded 217 records, in which 116 studies were included (Fig. 1). We did not identify extra eligible studies through manual searching. Finally, 116 studies were included in this meta-research study. The list of included studies, and the excluded studies with justifications are provided in Supplementary Datasheet.

### Adherence to sample size requirements

The topics and characteristics of the radiomics studies were heterogeneous (Table 1). There were 11/116 studies (9.5%) justified sample size for the model development [51–61], in which 6/116 (5.2%) performed a priori sample size calculation [53–55, 58, 60, 61], 2/116 (1.7%) applied the rule of thumb of EPV [56, 57], 1/116 (0.9%) conducted *post hoc* power analysis [59], 1/116 (0.9%) mentioned the sample size calculation but without results [52], and 1/116 (0.9%) mentioned the sample size justification but did not describe the methodology [51] (Supplementary Table S1).

**Table 1:** Study characteristics

Characteristics (*N* = 116)	Frequency (percentage)
Journal	
European Radiology	80 (69)
Insights into Imaging	25 (22)
Radiology	7 (6)
European Radiology Experimental	3 (3)
Radiology: Imaging Cancer	1 (1)
Radiology: Artificial Intelligence	0 (0)
Radiology: Cardiothoracic Imaging	0 (0)
Study design	
Retrospective	104 (90)
Prospective	12 (10)
Model testing method	
Development only or with cross-validation	11 (9)
Development with only internal testing	46 (40)
Development with only external testing	21 (18)
Development with internal and external testing	38 (33)
Imaging modality	
CT	54 (47)
MRI	49 (42)
PET	8 (7)
US	2 (2)
MMG	2 (2)
US and MMG	1 (1)
Specialty of topic	
Gastrointestinal	32 (28)
Neuro	18 (16)
Chest	11 (9)
Genitourinary	11 (9)
Breast	10 (9)
Cardiac and vascular	10 (9)
Obstetric/gynecologic	8 (7)
Head and Neck	7 (6)
Musculoskeletal	5 (4)
Forensic medicine	1 (1)
Intend use	
Diagnostic	81 (70)
Prognostic	35 (30)
Study consideration	
Presence of calibration metrics	68 (59)
Presence of sample size justification	11 (9)
Presence of sample size discussion	83 (72)
Reporting guideline	
RQS	6 (5)
TRIPOD	4 (3)
STARD	3 (3)
CLEAR	1 (1)
CLAIM	1 (1)

*RQS* radiomics quality score, *TRIPOD* transparent reporting of a multivariable prediction model for individual prognosis or diagnosis, *STARD* standards for reporting of diagnostic accuracy studies, *CLEAR* checklist for evaluation of radiomics research, *CLAIM* checklist for artificial intelligence in medical imaging

**Fig. 2:**
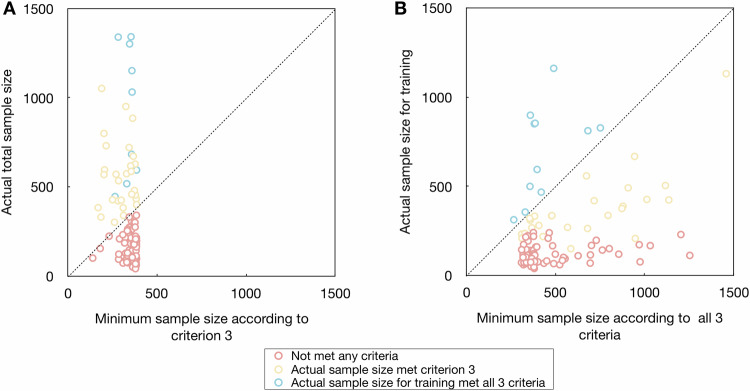
Adherence of actual sample size to minimum sample size requirements. Scatter plots of (**A**) actual total sample size and minimum sample size according to criterion 3, and (**B**) actual sample size for training and minimum sample size according to all three criteria. The red, yellow, and blue dots indicate studies with actual total sample size did not meet the minimum sample size requirements according to Riley et al criterion 3, studies with actual total sample size met the minimum sample size requirements according to Riley et al criterion 3, and studies with actual sample size for training met the minimum sample size requirements according to Riley et al all three criteria, respectively. The dashed line indicates the actual total sample size equals the minimum sample size requirements. The upper left area indicates the study met the minimum sample size requirements; the bottom right area indicates the study did not meet the minimum sample size requirements

The median (Q1, Q3) of the total sample size was 223 (130, 463) (Fig. 2 and Table 2). The median (Q1, Q3) of sample size for training, internal testing, and external testing were 150 (90, 288), 70 (35, 115), and 71 (45, 167), respectively. The median (Q1, Q3) of predictor parameters, events, and EPP were 14 (8, 20), 88 (57, 171), and 7.5 (4.3, 14.3), respectively.

**Table 2:** Summary of the sample size in the models

	Total sample size	Sample size for training	Sample size for internal testing	Sample size for external testing
Development only or with cross-validation, *n* = 11	74 (66, 116)	74 (66, 116)	n. a.	n. a.
Development with only internal testing, *n* = 46	185 (119, 291)	119 (85, 211)	54 (33, 88)	n. a.
Development with only external testing, *n* = 21	246 (154, 627)	193 (110, 335)	n. a.	65 (46, 183)
Development with internal and external testing, *n* = 38	443 (218, 658)	249 (121, 385)	90 (47, 166)	72 (45, 148)
Overall, *N* = 116	223 (130, 463)	150 (90, 288)	70 (35, 115)	71 (45, 167)

Data are present as median (Q1, Q3)

*n. a.* not applicable

**Fig. 3:**
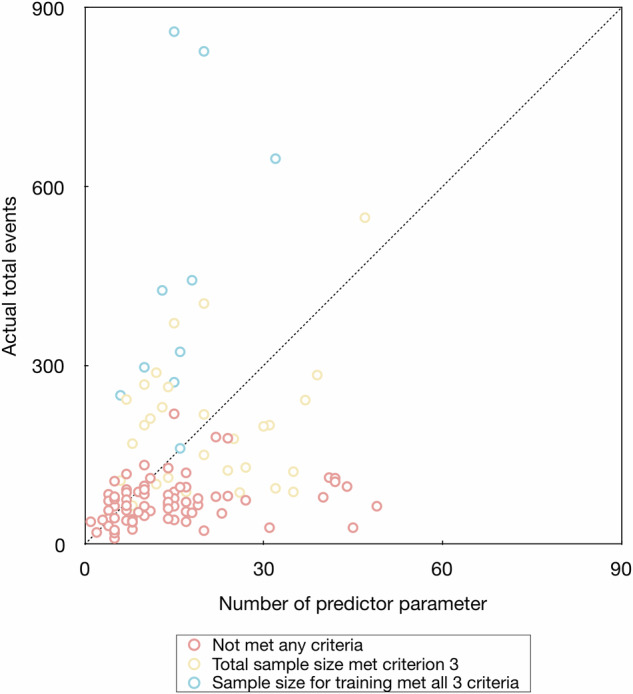
Comparison between the EPV ≥ 10 rule and Riley et al criteria. Scatter plot of a number of predictors in models against the actual number of events in model training. The red, yellow, and blue dots indicate studies with actual total sample size did not meet the minimum sample size requirements according to Riley et al criterion 3, studies with actual total sample size met the minimum sample size requirements according to Riley et al criterion 3, and studies with actual sample size for training met the minimum sample size requirements according to Riley et al all three criteria, respectively. The dashed line indicates the ten EPV rule. The upper left area indicates a study with EPV ≥ 10; the bottom right area indicates a study with EPV < 10

According to criterion 3 by Riley et al [41, 43, 45], the median (Q1, Q3) difference between the total sample size and minimum sample size was −100 (−216, 183); according to all three criteria by Riley et al [41, 43, 45], the median (Q1, Q3) of difference between the sample size for training and minimum sample size were −268 (−427, −157) (Fig. 2, Table 3, and Supplementary Fig. S1). There were 46, 29, and 19 studies met the EPV ≥ 10, EPV ≥ 15, and EPV ≥ 20 rule, respectively. The EPV ≥ 10 rule does not guarantee the sample size meets the minimum sample size requirements according to Riley et al (Fig. 3) [41, 43, 45]. There were six studies with sample size justification that met the weak requirement between total sample size and minimum required sample size according to criterion 3 [51, 53, 54, 57, 58, 60], and only one study with sample size justification met the strict requirement between sample size for training and minimum required sample size according to all three criteria [54].

**Table 3:** Adherence to requirements of sample size, number of events, and events per predictor parameter

	Sample size	Number of events	Events per predictor parameter
Minimum required sample size according to Riley et al			
Met criterion 1	339 (182, 547)	125 (70, 237)	8.8 (6.5, 13.2)
Met criterion 2	304 (178, 488)	114 (65, 175)	8.1 (6.8, 11.5)
Met criterion 3	349 (318, 376)	140 (101, 194)	10.7 (5.4, 17.4)
Met all three criteria	384 (358, 681)	173 (115, 247)	5.4 (8.7, 22.7)
Difference between total sample size and minimum required sample size according to criterion 3	−100 (−216, 183)	−34 (−99, 47)	−2.2 (−8.6, 2.9)
Difference between sample size for training and minimum required sample size according to all three criteria	−268 (−427, −157)	−102 (−180, −59)	−8.2 (−16.2, −3.7)

Data are present as median (Q1, Q3). The negative number indicates that the actual number did not meet the estimated minimum requirement

### Factors associated with sufficient sample size

There were 12/116 (10.3%) and 104/116 (89.7%) studies labeled as sufficient and insufficient for sample size, respectively, according to whether the sample size for training met the minimum required sample size calculated using all three criteria by Riley et al [41, 43, 45]. None of the investigated factors were associated with sufficient sample size (Supplementary Table S2). There were 42/116 (36.2%) and 74/116 (63.8%) studies labeled as sufficient and insufficient for sample size, respectively, according to whether the total sample size met the minimum required sample size calculated using criterion 3 by Riley et al (Supplementary Table S3) [41, 43, 45].

The univariable logistic regression analysis indicated that the journal, model testing type, and specialty of the topic were associated with sufficient sample size. The multivariable logistic regression analysis suggested that the studies with only external testing (OR 5.19, 95% CI: 1.32 to 20.39, *p* = 0.018) or with internal and external testing (OR 15.81, 95% CI: 4.43 to 56.45, *p* < 0.001) were more likely to have sufficient sample size than those with only internal testing. The studies of chest (OR 8.34, 95% CI: 1.55 to 44.92, *p* = 0.014), breast (OR 14.06, 95% CI: 1.78 to 111.36, *p* = 0.012), and cardiac and vascular (OR 20.39, 95% CI: 2.62 to 158.81, *p* = 0.004) were more likely to have sufficient sample size than those of gastrointestinal.

## Discussion

Our study found that less than one-tenth of the included radiomics studies justified the sample size. Our results were in accordance with previous studies that argued that the reporting of prediction models was lacking sample size calculation [62, 63]. Further, the sample size calculation was poorly reported and did not follow the guidance when reported [28, 36, 64]. Only six models in our study performed a priori sample size calculation. A previous study found that the rule of thumb of EPV and power analysis were usually used in the sample size of the prediction model [65]. In our study, five models applied power analysis [53, 55, 58, 60, 61], and only one model followed recommended approaches by Riley et al [54]. Further, it has been confirmed that the rule of thumb of EPV does not guarantee the sample size to meet the Riley et al criteria [50]. Therefore, we would like to recommend the radiomics studies to a priori calculate the minimum required sample size by Riley et al criteria to avoid the problem of overfitting [6, 23–26].

The sample size was insufficient in more than half of the included models when the weak requirement was applied with a median gap of 100 samples. Only 12 models were considered to be sufficient when the strict requirement was used; lacking samples with a median number of 268. Two studies in prostate cancer and binary outcome prediction models suggested that only 2% and 8% of models have reported a sample size calculation [50, 65], respectively, which is similar to the results in our study—5%. They reported 51% and 23% of models satisfying the Riley et al minimum sample size requirements [50, 65], while our study has a result of 40%. Although the percentage of the models with sufficient sample size varied among studies, there is no doubt that there remains a large room for improvement in sample size calculation in prediction model research. One major disadvantage of radiomics prediction models is that random chance associations may occur [24]. A sufficient sample is essential for the training and testing datasets. Our model found that models with a strategy of cross-validation usually had a smaller number of cases. These models should be interpreted with careful consideration of the danger of overfitting, leading to overestimation of model performance [24]. On the other hand, models with external testing are more likely to meet the sample size requirements. A stricter requirement on sample size may force the researchers to seek external testing of their models, and potentially improve the reproducibility and generalizability of models to other datasets, and translation into clinical practice [5, 26, 66].

The factors associated with sample size sufficiency when applying the strict requirement were not found. This can be attributed to insufficient positive outcomes in the regression analysis. The model testing type and specialty of the topic were found to be associated with sufficient sample size when applying the weak requirement. We supposed that the studies with external testing were performed with more consideration for methodology, and therefore, were more likely to have a sufficient sample size. One possible explanation is that chest, breast, and cardiac and vascular studies were more likely to have a sufficient sample size that they may find wider samples of patients as they may relate to more common pathologies or to diseases that get screened for or studied more often. However, it is unclear how to improve the sample size sufficiency in radiomics studies. As the radiomics community may not be familiar with the Riley et al sample size calculation criteria [40–46], our study may encourage the community to try the method, and test whether it is appropriate for radiomics research. Further, the recently published checklists emphasize the sample size in the study design and ask the authors to report the sample size calculation [15–17]. The checklists also support external testing of radiomics models, which is associated with a sufficient sample size. Moreover, the radiomics studies of chest, breast, and cardiac and vascular are more likely to have sufficient sample size. We hypothesized that these studies may benefit from large databases and clinical trial data in these fields [67–69]. Radiomics studies are expected to fulfill sample size requirements if the data-sharing practice is improved or incorporated into clinical trials [10, 24]. It is also necessary to emphasize the role of the journal in the sample calculation. We found that five out of seven included journals declared requirements for sample size justification in their submission guidelines, and these requirements were simple and sometimes not mandatory. We supposed that the unclear requirements for the sample size justification may lead to a smaller number of studies with sample size calculation, and heterogenous methodology for sample size or power calculation.

Our study has several limitations. First, only journals owned by the *European Society of Radiology* and the *Radiological Society of North America* were included in our study. We consider these leading peer-reviewed radiological journals may overestimate the radiomics studies with sample size justification. The publication time was also limited, since we decided to select recently published radiomics studies to present the latest situation in the field. Second, the number of candidate predictor parameters was not the true number of the candidate predictor parameters. The real number of features tested for model development is unknown. The non-linearity of the continuous variable resulted in more degrees of freedom, but this was not included in the calculation. The calculated minimum sample size was, therefore, potentially underestimated. Third, it is unclear how to improve the sample size sufficiency. As most of the included studies were published before the publication of radiomics checklists for reporting and methodology [15–17], the next work may explore if adherence would increase in the future. It is also interesting to investigate the relationship between the quality of the study and the presence of information on sample size justifications. Fourth, our study treated radiomics models as a kind of prediction model and focused on the sample size issue rather than specific methodological considerations of radiomics modeling. We believe the opportunities for carrying out reproducible and open research in the field of radiomics have already been well-described [10]. Fifth, our study only applied the Reily criteria for sample size calculation [40–46]. Compared with previous guidance around sample size requirements for prediction models, this method is tailored to the model to the model and clinical context-specific, and can ensure that the prediction model is robustly developed [23, 50, 65]. Nevertheless, there are other methods available for sample calculation for radiomics prediction models [70, 71], and some of them are also easy to use with website tools [71]. It still needs further investigation to determine which methods are appropriate for which situation. These investigations may be conducted in cooperation with methodologists but not only by radiologists. It would be interesting for the radiomics community to compare the results of different methods and later reach a consensus. Finally, this study focused on the radiomics prediction models for binary outcomes. As such, one could argue that the findings might not generalize to prediction models using deep learning methods, or time-to-event and continuous outcomes.

In conclusion, this study suggested that radiomics studies were often designed without sample size justification or calculated with inappropriate methods. The sample size of radiomics models is usually too small to avoid overfitting. Sample size calculation with criteria proposed by Riley et al is encouraged when developing a radiomics model.

## Supplementary information

ELECTRONIC SUPPLEMENTARY MATERIAL

Datasheet

## Abbreviations

EPP: Events per predictor parameter
EPV: Events per variable
Q1: First quartile
Q3: Third quartile
