# Supplementary material for: Overlooked and underpowered: a meta-research addressing sample size in radiomics prediction models for binary outcomes
Source: Eur Radiol. 2025 Jan 9;35(3):1146–56. doi: 10.1007/s00330-024-11331-0 (PMC11835977; doi:10.1007/s00330-024-11331-0)

Overlooked and underpowered: a meta-research addressing sample size in radiomics prediction models for binary outcomes
ELECTRONIC SUPPLEMENTARY MATERIAL

List of Supplementary Material

Supplementary Note S1 Study protocol
Supplementary Note S2 Search strategy and study selection
Supplementary Note S3 Data extraction and management
Supplementary Note S4 Data analysis

Supplementary Table S1 Sample size justification in radiomics studies
Supplementary Table S2 Factors associated with sufficient sample size according the weak requirement
Supplementary Table S3 Factors associated with sufficient sample size according the strict requirement

Supplementary Figure S1 Adherence to sample size requirements

Supplementary Note S1 Study protocol
First drafted date: 10 December 2023
Last modification date: 26 December 2023

Study rationale
Radiomics is a popular model building approach in the radiological field. Although sample size is mentioned in radiomics and artificial intelligence reporting guidelines, it is seemingly rarely reported in radiomics studies. It is unclear if and how the sample size is determined in radiomics studies. In this study, we aimed to (1) evaluate the if and how sample sizes were justified in radiomics models; and (2) calculate the minimum sample size required for each radiomics model, and compare it with the actual sample size used.

Study design
This is a meta-research study that focuses on the methodological aspect of the published literatures on radiomics models. Institutional ethical approval or written informed consent are not required because there are no animal or human subjects that have been included in the study.

Study selection
Our study will include primary studies developing a radiomics prediction model for a binary, patient-related health outcome published online between 01 January 2023 and 31 December 2023 in leading radiology journals. The studies will be excluded if they were: (a) duplicates; (b) not primary study; (c) study developing models using deep learning methods; (d) study predicting time-to-event or continuous outcomes; (e) model validation only study or study not aimed to develop prediction models; (f) study with insufficient data or full-text not available. The results of study selection will be cross-checked.

Data extraction
Our review group will develop a standardized data extraction sheet for general information (journal, first author, imaging modality, specialty of topic, intend use, and applied reporting guideline), model development methodology (model testing method, study design, presence of sample size calculation or justification, presence of discussion on sample size, and presence of calibration metrics), and model metrics for sample size calculation (sample size, number of events, number of predictor parameters, Cox-Snell R-square value or the c-statistic if the R-square value was not reported). The results of data extraction will be cross-checked.

Data analysis
The data analysis will be carried out using R language. The statistical analysis will be performed using *DescTools* package, and the calculation of minimum sample size will be conducted using *pmsampsize* package. The event per predictor parameter (EPP) will be calculated as EPP = the number of events/ the number of candidate predictor parameters. We consider the number of parameters in the final model for the calculations since there are usually hundreds of radiomics features extracted from the images. It is nonsense to use the number of radiomics features for calculation before feature dimension reduction. The sample sizes will be calculated using the Riley et al. formulae according to 3 criteria, respectively. The study will be labeled as sufficient for sample size if the sample size for developing met the minimum required sample size according to Riley et al. criterion 3; otherwise, it will be labeled as insufficient for sample size. The study characteristics will be evaluated by logistic regression analysis to tell whether they are associated with the sufficiency of sample size.

Reporting and dissemination
We plan to report this study via peer-reviewed journals. One member will draft the original version of the manuscript. All the members will read and edit the manuscript critically. We plan to disseminate our study via conference abstracts, journal articles, and oral presentations.

Supplementary Note S2 Search strategy and study selection

Our study developed a search strategy that covers all the official journal of the European Society of Radiology and Radiological Society of North America. These journals are also indexed with high impact factors in the Science Citation Index Expanded (SCIE) or Emerging Science Citation Index (ESCI), Radiology, Nuclear Medicine & Medical Imaging category, 2022 Journal Citation Reports. We believe this strategy can retrieved representative high-quality radiomics studies for our meta-research study. The volume of the retrieved records is a reasonable number, to allow us to assess them in detail.

1. Preliminary search
The following search strategies are tested for feasibility for our study. The pilot search is performed on 31 December 2023.

(1) European Radiology + Radiology
Search string:
("radiomics"[Title/Abstract] OR "radiomic"[Title/Abstract]) AND ("Radiology"[Journal] OR "European Radiology"[Journal]) AND (2023/01/01:2023/12/31[Date - Publication]) NOT ("Review"[Publication Type] OR "Systematic Review"[Publication Type] OR "Editorial"[Publication Type] OR "Congress"[Publication Type] OR "Comment"[Publication Type] OR "Published Erratum"[Publication Type] OR "Letter"[Publication Type])
Retrieved records:
170
Comment:
The volume of these two journals is limited. There are many radiomics studies published on the Radiology Artificial Intelligence journal and Insights into Imaging journal. Although some of them are not in the Science Citation Index Expanded (SCIE), Radiology, Nuclear Medicine & Medical Imaging category, 2022 Journal Citation Reports, studies are published on these journals are also with high quality. Some of these journals are already given an impact factor and in the Emerging Science Citation Index (ESCI), Radiology, Nuclear Medicine & Medical Imaging category, 2022 Journal Citation Reports. We believe the included journals should be extended.

(2) ESR + RSNA journals
Search string:
("radiomics"[Title/Abstract] OR "radiomic"[Title/Abstract]) AND ("Radiology"[Journal] OR "Radiology Artificial Intelligence"[Journal] OR "Radiology Imaging Cancer"[Journal] OR "Radiology Cardiothoracic Imaging" [Journal] OR "European Radiology"[Journal] OR "Insights into Imaging"[Journal] OR "European Radiology Experimental"[Journal]) AND (2023/01/01:2023/12/31[Date - Publication]) NOT ("Review"[Publication Type] OR "Systematic Review"[Publication Type] OR "Editorial"[Publication Type] OR "Congress"[Publication Type] OR "Comment"[Publication Type] OR "Published Erratum"[Publication Type] OR "Letter"[Publication Type])
Retrieved records:
217
Comment:
This strategy covers the official journals of European Society of Radiology and Radiological Society of North America. These journals are also indexed with high impact factors in the Science Citation Index Expanded (SCIE) or Emerging Science Citation Index (ESCI), Radiology, Nuclear Medicine & Medical Imaging category, 2022 Journal Citation Reports. We believe this strategy can retrieved representative high-quality radiomics studies for our meta-research study. The volume of the retrieved records is a reasonable number, to allow us to assess them in detail.

(3) Strategy extracted from "Towards reproducible radiomics research: introduction of a database for radiomics studies"
Citation:
Akinci D'Antonoli T, Cuocolo R, Baessler B, Pinto Dos Santos D. Towards reproducible radiomics research: introduction of a database for radiomics studies. Eur Radiol. 2023 Aug 12. doi: 10.1007/s00330-023-10095-3. Epub ahead of print. PMID: 37572188.
Search string:
("radiomics"[Title/Abstract] OR "radiomic"[Title/Abstract]) AND ("Radiology"[Journal] OR "Radiology Artificial Intelligence"[Journal] OR "Radiology Imaging Cancer"[Journal] OR "European Radiology"[Journal] OR "European Journal of Radiology"[Journal]) AND (2023/01/01:2023/12/31[Date - Publication]) NOT ("Review"[Publication Type] OR "Systematic Review"[Publication Type] OR "Editorial"[Publication Type] OR "Congress"[Publication Type] OR "Comment"[Publication Type] OR "Published Erratum"[Publication Type] OR "Letter"[Publication Type])
Retrieved records:
205
Comment:
As described in the article, "We randomly selected leading first quarter (Q1) journals from Europe and the USA, namely European Radiology, European Journal of Radiology, Radiology, Radiology: Artificial Intelligence, Radiology:

Cardiothoracic Imaging, and Radiology: Imaging Cancer." However, European Journal of Radiology is a Q2 journal. Further, not all ESR and RSNA journals are included. It is unclear what the criteria are for the journal selection.

(4) Strategy extracted from "NEgatiVE results in Radiomics research (NEVER): A meta-research study of publication bias in leading radiology journals"

Citation:

Kocak B, Bulut E, Bayrak ON, Okumus AA, Altun O, Borekci Arvas Z, Kavukoglu I. NEgatiVE results in Radiomics research (NEVER): A meta-research study of publication bias in leading radiology journals. Eur J Radiol. 2023 Jun;163:110830. doi: 10.1016/j.ejrad.2023.110830. Epub 2023 Apr 11. PMID: 37119709.

Search string:

("radiomics"[Title/Abstract] OR "radiomic"[Title/Abstract]) AND ("Academic Radiology"[Journal] OR "American Journal of Neuroradiology"[Journal] OR "AJR American Journal of Roentgenology"[Journal] OR "Diagnostic and Interventional Imaging"[Journal] OR "European Journal of Radiology"[Journal] OR "European Radiology"[Journal] OR "Insights into Imaging"[Journal] OR "International Journal of Computer Assisted Radiology and Surgery Investigative Radiology"[Journal] OR "Journal of Magnetic Resonance Imaging"[Journal] OR "Journal of Neuroradiology"[Journal] OR "Journal of Vascular and Interventional Radiology"[Journal] OR "Korean Journal of Radiology"[Journal] OR "La Radiologia Medica"[Journal] OR "Radiology"[Journal]) AND (2023/01/01:2023/12/31[Date - Publication]) NOT ("Review"[Publication Type] OR "Systematic Review"[Publication Type] OR "Editorial"[Publication Type] OR "Congress"[Publication Type] OR "Comment"[Publication Type] OR "Published Erratum"[Publication Type] OR "Letter"[Publication Type])

Retrieved records:

402

Comment:

As described in the article, "The search was limited to original research studies published in Q1 (first quartile) clinical radiology journals according to SCImago Journal Rank based on Scopus (Elsevier, Netherlands) and related to radiology, nuclear medicine, and imaging. An additional filter was also applied to select journals that are also indexed in the Web of Science (Clarivate Analytics, London, United Kingdom). The search syntax was created by combining two components: radiomics-related terms and journal titles. Our group identified clinical radiology journals based on their academic background." It is unclear what the academic background of the journals is. Further, there are no radiomics articles identified in some the included journals: "American Journal of Neuroradiology", "International Journal of Computer Assisted Radiology and Surgery Investigative Radiology", "Journal of Magnetic Resonance Imaging", "Journal of Neuroradiology", "Journal of Vascular and Interventional Radiology".

(5) Strategy extracted from "Publications poorly report the essential RadiOmics ParametERs (PROPER): A *meta*-research on quality of reporting"

Citation:

Kocak B, Yuzkan S, Mutlu S, Bulut E, Kavukoglu I. Publications poorly report the essential RadiOmics ParametERs (PROPER): A meta-research on quality of reporting. Eur J Radiol. 2023 Oct;167:111088. doi: 10.1016/j.ejrad.2023.111088. Epub 2023 Sep 9. PMID: 37713968.

Search string:

(((((((radiomics) NOT (deep)) NOT (deep learning)) NOT (commentary)) NOT (editorial)) NOT (systematic review)) NOT (review)) NOT (editorial comment) AND (2023/01/01:2023/12/31[Date - Publication]) NOT ("Review"[Publication Type] OR "Systematic Review"[Publication Type] OR "Editorial"[Publication Type] OR "Congress"[Publication Type] OR "Comment"[Publication Type] OR "Published Erratum"[Publication Type] OR "Letter"[Publication Type])

Retrieved records:

1984

Comment:

The journal restriction is not applied. The study randomly chose 87 articles for investigation. This strategy is not suitable for our study.

(6) Strategy extracted from "Meta-research: How many diagnostic or prognostic models published in radiological journals are evaluated externally?"

Citation:

Hameed M, Yeung J, Boone D, Mallett S, Halligan S. Meta-research: How many diagnostic or prognostic models published in radiological journals are evaluated externally? Eur Radiol. 2024 Apr;34(4):2524-2533. doi: 10.1007/s00330-023-10168-3. Epub 2023 Sep 12. PMID: 37696974; PMCID: PMC10957714.

Search string:

("radiomics"[Title/Abstract] OR "radiomic"[Title/Abstract]) AND ("Radiology"[Journal] OR "European Radiology"[Journal] OR "Investigative Radiology"[Journal]) AND (2023/01/01:2023/12/31[Date - Publication]) NOT ("Review"[Publication Type] OR "Systematic Review"[Publication Type] OR "Editorial"[Publication Type] OR "Congress"[Publication Type] OR "Comment"[Publication Type] OR "Published Erratum"[Publication Type] OR "Letter"[Publication Type])

Retrieved records:

177
Comment:
The journal *Investigative Radiology* could be an important source of high-quality radiomics study.

2. Formal search

(1) PubMed search

Two independent reviewers (JYZ and XWL) run the search strategy on 01 Jan 2024 via the PubMed database (www.pubmed.ncbi.nlm.nih.gov). We only use one information source since it is unlikely to change the results by using multiple information sources, as all the papers on seven selected leading peer-reviewed journals will be indexed in PubMed.

The following is the search results via PubMed.

No	Search string	Aim	Retrieved records
#1	"radiomics"[Title/Abstract] OR "radiomic"[Title/Abstract]	To identify records relevant to radiomics	9,990
#2	"Radiology"[Journal] OR "Radiology Artificial Intelligence"[Journal] OR "Radiology Imaging Cancer"[Journal] OR "Radiology Cardiothoracic Imaging" [Journal] OR "European Radiology"[Journal] OR "Insights into Imaging"[Journal] OR "European Radiology Experimental"[Journal]	To identify records published on seven selected leading radiological journals	55,700
#3	2023/01/01:2023/12/31[Date - Publication]	To identify records published between 2023/01/01 and 2023/12/31	1,656,356
#4	"Review"[Publication Type] OR "Systematic Review"[Publication Type] OR "Editorial"[Publication Type] OR "Congress"[Publication Type] OR "Comment"[Publication Type] OR "Published Erratum"[Publication Type] OR "Letter"[Publication Type]	To identify records that are not original research articles	5,764,823
#5	#1 AND #2 AND #3 NOT #4	The final search	217

The complete search string:
("radiomics"[Title/Abstract] OR "radiomic"[Title/Abstract]) AND ("Radiology"[Journal] OR "Radiology Artificial Intelligence"[Journal] OR "Radiology Imaging Cancer"[Journal] OR "Radiology Cardiothoracic Imaging" [Journal] OR "European Radiology"[Journal] OR "Insights into Imaging"[Journal] OR "European Radiology Experimental"[Journal]) AND (2023/01/01:2023/12/31[Date - Publication]) NOT ("Review"[Publication Type] OR "Systematic Review"[Publication Type] OR "Editorial"[Publication Type] OR "Congress"[Publication Type] OR "Comment"[Publication Type] OR "Published Erratum"[Publication Type] OR "Letter"[Publication Type])

(2) Manual search

Further, two independent reviewers (XY and YFH) performed extra manual searched via the homepages of seven radiological journals (*European Radiology,* https://link.springer.com/journal/330; *Insights into Imaging,* https://insightsimaging.springeropen.com; *European Radiology Experimental*, https://eurradiolexp.springeropen.com; *Radiology*, https://pubs.rsna.org/journal/radiology; *Radiology: Artificial Intelligence*, https://pubs.rsna.org/journal/ai; *Radiology: Cardiothoracic Imaging*, https://pubs.rsna.org/journal/cardiothoracic; and *Radiology: Imaging Cancer,* https://pubs.rsna.org/journal/imaging-cancer) on 15 Jan 2024 to identify additionally potentially eligible study. There was no additionally potentially eligible study identified via the manual search of homepages of seven radiological journals on 15 Jan 2024.

Supplementary Note S3 Data extraction and management

Our review group developed a standardized data extraction sheet. One reviewer (JYZ) extracted the data from all included studies using the standardized data extraction sheet, and the results were double-checked by another reviewer (XWL, YX, HFY, DFD, or XG). Disagreements were discussed and resolved between the two reviewers, or adjudicated by our review group if necessary. The following is the data items and explanations.

(1) General information
Journal: Which journal that the article is published. This item can be directly retrieved via the PubMed.
First author: Who is the first author of the article. This item can be directly retrieved via the PubMed.
Imaging modality: The imaging modality of the radiomics study, such as radiography or mammography (MMG), computed tomography (CT), magnetic resonance imaging (MRI), positron emission tomography (PET), and ultrasound (US). If study use multiple imaging modalities, all of them are recorded, e. g., CT and MRI.
Specialty of topic: What is the subspecialty of this radiomics study, such as breast, cardiac and vascular, chest, gastrointestinal, genitourinary, head and neck, musculoskeletal, neuro, obstetric/gynecologic, pediatrics, etc.
Intend use: The models are classified by the intend use into diagnostic, and prognostic. The diagnostic models are prediction models that estimate the probability or risk that a specific disease or condition is present, while the prognostic models are or prediction models that estimate a specific event will occur in the future. If the study established prediction models for both diagnostic and prognostic purpose, both of them are recorded, i. e., diagnostic, and prognostic.
Applied reporting guideline: Whether the study applied a reporting guideline, and what is the reporting guideline, such as general reporting guidelines including CONSORT (Consolidated Standards of Reporting Trials) for randomized trials, STROBE (STrengthening the Reporting of Observational Studies in Epidemiology) for observational studies, STARD (Standards for Reporting of Diagnostic Accuracy) for diagnostic/prognostic studies, and TRIPOD (Transparent Reporting of a multivariable prediction model for Individual Prognosis Or Diagnosis) for diagnostic/prognostic studies; or reporting guidelines for radiomics studies including RQS (Radiomics Quality Score), IBSI (Image Biomarker Standardization Initiative), and CLEAR (CheckList for EvaluAtion of Radiomics research).

(2) Model development methodology
Model testing method: whether the study is development of model with/without internal/external testing. The sample size for training, and testing are defined as the sample used to train the model, and the sample used to test the model. The sample size for internal testing, and external testing are defined as the sample used to test the model from the same source of sample size for training, and the sample used to test the model from geographically different source of sample size for training. The testing with randomly data splitting, cross-validation, bootstrapping approaches are not considered as external testing.
Study design: whether the study is prospectively or retrospectively designed.
Presence of sample size calculation or justification: whether the study mentioned the sample size calculation or justification in the method section as a part of study design.
Presence of discussion on sample size: whether the sample size calculation or justification was discussed, such as recognition of a small sample size in the limitation section.
Presence of calibration metrics: whether the calibration metrics (such as calibration curve, or Hosmer-Lemeshow test) were present in the study.

(3) Model metrics for sample size calculation
Sample size: The sample size for training, and testing are defined as the sample used to train the model, and the sample used to test the model. The sample size for internal testing, and external testing are defined as the sample used to test the model from the same source of sample size for training, and the sample used to test the model from geographically different source of sample size for training. The testing with randomly data splitting, cross-validation, bootstrapping approaches are not considered as external testing.
Number of events and prevalence: The number of events is defined as the number of cases that the prediction model aimed to predict. The prevalence of the case is calculated as the number of events / the number of total sample size. We only calculated the prevalence of the whole study since it can better present the prevalence of the disease status than that in developing, internal testing, or external testing dataset. The number of events here would be used to calculate the event per predictor parameter. The prevalence here is necessary and would be used to calculate the required sample size.
Number of predictors parameters: We considered the number of predictor parameters in the final radiomics model for the calculation, i.e., the number of radiomics and non-radiomics features that are selected to build the final model. As hundreds of radiomics features would be extracted from the images during the radiomics workflow, it is nonsense to use the number of extracted features as the candidate predictor parameters for calculation. However, it would be helpful to *post hoc* check whether the sample size for developing the radiomics model is enough to allow the number of features selected, or to *a priori* calculate how many features the sample size can support.
Cox-Snell R^2 value or the C-statistic if the R^2 value was not reported: the Cox-Snell R-square value is necessary for the calculation of the required sample size. However, in most cases, the Cox-Snell R-square value is not reported

in radiomics studies. It is possible to the Cox-Snell R-square value from published studies if they are not reported, using other information, such as the C-statistic or Nagelkerke's R-square value (Stat Med, 2021;40(4):859-864). Therefore, we recorded the c-statistic instead, which is usually reported to represent the performance of the radiomics model. The calculation can be realized using the pmsampsize package in R language. If there are multiple models built in a study, the one with highest C-statistics is included. If there are multiple models with the same high C-statistics, the one with higher Youden index is included. This model is most likely to be translate to clinical practice. If there are C-statistics for the developing, internal testing, and external testing of datasets, respectively, the C-statistics for external testing dataset is included. It can most accurately and objectively represent the performance of the model.

Supplementary Note S4 Data analysis

The data analysis was carried out using R language version 4.1.3 within RStudio version 1.4.1106. The statistical analysis was performed using *DescTools* package version 0.99.54 (https://cran.r-project.org/web/packages/DescTools/index.html), and the calculation of minimum sample size was conducted using *pmsampsize* package version 1.1.3 (https://cran.r-project.org/web/packages/pmsampsize/index.html). All the statistical analysis was two-sided and the alpha level for statistically significance is set at 0.05, unless declared otherwise. The sample sizes were presented as mean ± standard deviation (SD), median (first and third quartile, Q1 and Q3). Word cloud was generated using a free online tool (WordClouds, Zygomatic; https://www.wordclouds.com) to visualize the topics of the radiomics studies.

The *pmsampsize* package is summarized to compute the minimum sample size required for the development of a new multivariable prediction model with continuous, binary or survival (time-to-event) outcomes using the criteria proposed by Riley et al. (Stat Methods Med Res. 2019;28(8):2455-2474; Stat Med, 2019;38(7):1262-1275; Stat Med, 2019;38(7):1276-1296). These aim to minimize the overfitting and to ensure precise estimation of key parameters in the prediction model. For continuous outcomes, there are four criteria: (1) small overfitting defined by an expected shrinkage of predictor effects by 10% or less, (2) small absolute difference of 0.05 in the model's apparent and adjusted R-squared value, (3) precise estimation of the residual standard deviation, and (4) precise estimation of the average outcome value. The sample size calculation requires the user to pre-specify (e. g. based on previous evidence) the anticipated R-squared of the model, and the average outcome value and standard deviation of outcome values in the population of interest. For binary or survival (time-to-event) outcomes, there are three criteria: (1) small overfitting defined by an expected shrinkage of predictor effects by 10% or less, (2) small absolute difference of 0.05 in the model's apparent and adjusted Nagelkerke's R-squared value, and (3) precise estimation (within +/- 0.05) of the average outcome risk in the population for a key timepoint of interest for prediction. In the current study, we applied the criteria for binary outcomes, i. e.: (1) small overfitting defined by an expected shrinkage of predictor effects by 10% or less, (2) small absolute difference of 0.05 in the model's apparent and adjusted Nagelkerke's R^2 value, and (3) precise estimation (within +/- 0.05) of the average outcome risk in the population.

The event per predictor parameter (EPP) was calculated as EPP = the number of events/ the number of candidate predictor parameters (J Clin Epidemiol, 2021; 133:53-60; BMC Med Res Methodol, 2023;23(1):188). We considered the number of parameters in the final model for the calculations since there are usually hundreds of radiomics features extracted from the images. It is of nonsense to use the number of radiomics features for calculation before feature dimension reduction, or the minimum sample size will be always dozens of thousands of events that can never be satisfied. The sample sizes were calculated using the Riley et al. formulae according to 3 criteria, respectively (Stat Methods Med Res. 2019;28(8):2455-2474; Stat Med, 2019;38(7):1262-1275; Stat Med, 2019;38(7):1276-1296; Stat Med, 2021;40(1):133-146; Stat Med, 2021;40(19):4230-4251; Stat Methods Med Res, 2023;32(3):555-571). The minimum sample size that meets all 3 criteria needed to be not smaller than the largest of the three calculated sample sizes. The calculation was based on the sample size and number of events used for developing model, the number of predictors in the model, and the Cox-Snell R^2 value or the c-statistic (Stat Med, 2021;40(4):859-864). The criterion 3 were separately assessed because this criterion can be considered the absolute lowest sample size that could be accepted when developing a prediction model (J Clin Epidemiol, 2021; 133:53-60; BMC Med Res Methodol, 2023;23(1):188). We later calculated the difference between total sample size and minimum required sample size according to Riley et al criterion 3, and the difference between sample size for training and minimum required sample size according to Riley et al all 3 criteria, respectively. The former one is a relatively weak requirement that is easier to achieve, while the later one is a relative strict requirement.

```
-------------------------------------------------------------------------
## Example of pmsampsize package based on fictional data

## Binary outcomes (Logistic prediction models)
## Use pmsampsize to calculate the minimum sample size required to develop
## a multivariable prediction model for a binary outcome using 10 radiomics features
## Based on the data in the study, the outcome prevalence is anticipated to be 56/258
## = 0.217 (21.7%) and the Cox-Snell R-squared the existing prediction model is not
## reported. Instead, we recorded a C-statistic (AUC) of the external testing model of 0.85.
## We can use this C-statistic along with the prevalence to approximate the Cox-Snell
## R-squared value. We can use pmsampsize with the cstatistic() option instead of rsquared() option.

## Then we write the following code
pmsampsize(type = "b", cstatistic = 0.85, parameters = 10, prevalence = 0.217)

## The following is the return results
## Given input C-statistic = 0.85 & prevalence = 0.217
```

```
## Cox-Snell R-sq = 0.2616
## NB: Assuming 0.05 acceptable difference in apparent & adjusted R-squared
## NB: Assuming 0.05 margin of error in estimation of intercept
## NB: Events per Predictor Parameter (EPP) assumes prevalence = 0.217
##            Samp_size Shrinkage Parameter CS_Rsq Max_Rsq Nag_Rsq EPP
## Criteria 1       292      0.90        10 0.2616   0.649   0.403 6.34
## Criteria 2       261      0.89        10 0.2616   0.649   0.403 5.66
## Criteria 3       262      0.90        10 0.2616   0.649   0.403 5.69
## Final            292      0.90        10 0.2616   0.649   0.403 6.34
## Minimum sample size required for new model development based on user inputs = 292,
## with 64 events (assuming an outcome prevalence = 0.217) and an EPP = 6.34

## The minimum sample size for all the models is calculated use pmsampsize package.
```

The study would be labeled as sufficient for sample size according to (1) a relatively weak requirement: if the sample size for training met the minimum required sample size according to Riley et al all 3 criteria; or (2) a relatively strict requirement: the total sample size and minimum required sample size according to Riley et al criterion 3. The study characteristics were evaluated by logistic regression analysis to tell whether they are associated with the sufficiency of sample size: (1) journal, (2) imaging modality, (3) specialty of topic, (4) intend use, (5) whether the study applied reporting guideline, (6) model testing method, (7) study design, (8) presence of sample size calculation or justification, (9) presence of discussion on sample size, and (10) presence of calibration metrics. These factors are firstly tested using univariate logistic regression to tell whether they are associated with the sufficient sample size, with an alpha level of 0.10. Multiple logistic regression analysis is used to estimate adjusted odds ratio (OR) and 95% confidential interval (CI) of whether factors are associated with the sufficient sample size, with an alpha level of 0.05.

Supplementary Table S1 Sample size justification method in radiomics studies

Author	Sample size justification	Sample size calculation	Sample size justification method	Ref
Zhao HP	Yes	No	In terms of the sample size estimating, we balanced the efficiency of model building and the generalization ability of the results to the greatest extent, based on the explanations of the TRIPOD statement.	[53]
Chen YY	Yes	Yes, but the results are not present.	The sample size calculation criteria were as follows: 80% power, AUC = 0.80, two-sided significance level set at 0.05, with an allocation ratio of 1.	[54]
Lin ZJ	Yes	Yes	For the training cohort, the assumptions made during the sample size calculation were case-to-noncase ratio of 1:3, desired AUC of 0.85, confidence internal width of 0.15, and the two-sided α level of 0.05. Without considering the design effect of the dropout rate, the total sample size was 180 with 45 recurrence cases and 135 non-recurrence cases. Therefore, we believed that the sample size of our model in the training cohort (235 with 56 recurrence cases and 179 non-recurrence cases) can meet the statistical test efficiency.<br>For the validation cohorts, Shein-Chung Chow and colleagues introduced a significance level (α=0.05), power (1-β=0.95), case-to-noncase ratio (1:3), means and standard deviation referring the results in the training cohort, the sample size needed in the validation cohorts was calculated to be 16 with recurrence and 48 with non- recurrence. In our study, 102 (recurrence: 24, non-recurrence: 78) and 84 (recurrence: 21, non-recurrence: 63) patients were respectively included in the internal and external cohorts, which were exceeding the minimum required sample sizes.	[55]
Zysman M	Yes	Yes	A sample size of the development cohort was calculated using Riley and colleagues' approach. We hypothesized an incidence of significant clinical deterioration within 30 days at 20%; among mild COVID-19, 16 parameters included in the clinical prediction models and an expected Harrell's c-index of 0.78 (Nagelkerke's R2 of 0.25). The resulting sample size was at least 826 patients. For the external validation, we aimed to recruit at least one hundred clinical deterioration events for each validation cohort, as recommended by Vergouwe.	[56]
Wang P	Yes	Yes	The study consecutively enrolled approximately 70 subjects. Regarding AUC (H1), 70 subjects were needed for the study to detect an AUC of 0.80 for ADGGIP at ~85% power and alpha = 0.05 (two-sided). The sample size calculation was based on the following assumptions: 1) the AUC in the null hypothesis was 0.60, and 2) the positive cases accounted for 66% of the population.<br>The sample size and power calculations were performed in PASS 2021 software.	[57]
Wang J	Yes	No, but used the rule of thumb of ten outcome events per variable.	During the RFE feature selection, we set the feature number from 3 to 12 (the maximum feature number should not exceed 1/10 of sample size; otherwise, the model would overfit), respectively.	[58]
Feng CJ	Yes	No, but used the rule of thumb of ten outcome events per variable.	Given the sample size of 280 patients in the training cohort, we chose the 10 most important features to build the optimal random forest model by adjusting model parameters through grid search	[59]
Ramtohul T	Yes	Yes	Given the null hypothesis of an AUC of 0.50 and the alternate hypothesis of an AUC of 0.70, the proportion of 75% and 25% HER2-low and -positive to HER2- zero cancers, and a statistical power of 90%, we calculated that the minimum required sample of patients in the external test set was 124.	[60]

Sun JJ	Yes	Yes, *post hoc* power calculation.	Considering the relatively small sample size, effect size and confidence interval were calculated to measure the practical significance. The result showed a 0.8 effect size was detected with 98.1% confidence, a maximum of 5% probability of misreporting differences. Moreover, the value of empirical effect size was calculated (d = − 2.504). The result showed that the effect size between the two groups was large and the difference in the mean between the two groups was significant, which indicated that the empirical sample size included in this study could support the conclusion.	[61]
Xiao ML	Yes	Yes	Primary cohort: We retrospectively chose 150 patients treated in Center 1 between January 2010 and December 2015 as the primary cohort. When the case-to-noncase ratio was 1:1, logistic regression performed best. To balance the training samples and make the case-to-noncase ratio close to 1:1, we designed the primary cohort to contain 50 PMI-positive ASC/AC patients, 50 PMI-negative AC patients, and 50 PMI-negative ASC patients who were randomly selected from the 133 PMI-negative patient pool. To avoid model overfitting, the principle that radiomics features were less than 1/8 of the training sample size was used. In our study, 9 intratumoral original and wavelet (Ori-Wav) features and 10 peritumoral Ori-Wav features were selected to build the RST and RS5 for predicting PMI in cervical AC/ASC patients. The primary sample size was 150. Therefore, based on the above principle, the relationship between the number of patients in the primary cohort and the selected features for the radiomics signature was acceptable.<br>Validation cohorts: We calculated the necessary sample size of labelled PMI-positive for the internal and external validation cohorts. We calculated the sample size based on a type I error of 5% with 80% power. We assumed the null hypothesis with an AUC of 0.70, an alternative hypothesis with an AUC of 0.90, and a ratio of negative to PMI of 6:1, yielding a sample of at least 9 PMI patients needed. Power calculation was performed using MedCalc (Version 19.6.4, MedCalc Software Ltd).	[62]
Xu Y	Yes	Yes	The sample size calculation. The sample size was calculated with the PASS software 2021, v21.0.3. The median survival time was 46.6 and 9.6 months in the treatment (TLSs-positive) and control (TLSs-negative) groups which based on the training cohort data, with alpha of 0.05 and beta of 0.1952. The power was 0.80 and alternative hypothesis was one-sided. Based on the above real-world data in the training cohort, the ratio of positive to negative group was 1:2, and we set the positive: negative group as 1:2. Finally the sample size should be 10 in the TLSs-positive group and 20 in the TLSs-negative. And this is the reason why we included the 30 patients as the external validation cohort.	[63]

Supplementary Table S2 Factors associated with sufficient sample size according to the weak requirement

Variable grouping	Univariable logistic analysis			Multivariable logistic analysis		
	OR	95% CI	P value	OR	95% CI	P value
Journal						
European Radiology	Ref		0.158	n. a.		
Insights into Imaging	1.682	0.388-7.281	0.487			
Other	4.625	0.966-22.145	0.055			
Study design						
Retrospective	Ref		0.290	n. a.		
Prospective	0.516	0.151-1.759	0.290			
Model testing method						
Development with only internal testing	Ref		0.597	n. a.		
Development only or with cross-validation	1.433	0.135-15.264	0.765			
Development with only external testing	1.509	0.233-9.778	0.666			
Development with internal and external testing	2.687	0.624-11.566	0.184			
Imaging modality						
CT	Ref		0.352	n. a.		
MRI	0.375	0.094-1.503	0.166			
Other	0.479	0.055-4.213	0.507			
Specialty of topic						
Gastrointestinal	n. a.			n. a.		
Neuro						
Chest						
Genitourinary						
Breast						
Cardiac and vascular						
Other						
Intend use						
Prognostic	Ref		0.681	n. a.		
Diagnostic	0.750	0.190-2.956	0.681			
Calibration metrics						
Absence	Ref		0.983	n. a.		
Presence	0.987	0.294-3.317	0.983			
Sample size justification						
Absence	Ref		0.886	n. a.		
Presence	0.855	0.100-7.325	0.886			
Sample size discussion						
Absence	Ref		0.290	n. a.		
Presence	0.516	0.151-1.759	0.290			
Reporting guideline						
None	Ref		0.685	n. a.		
Applied	1.400	0.276-7.114	0.685			

The study would be labeled as sufficient for sample size according to (1) a relatively weak requirement: if the sample size for training met the minimum required sample size according to Riley et al all 3 criteria; or (2) a relatively strict requirement: the total sample size and minimum required sample size according to Riley et al criterion 3. There are 12 and 104 studies are labeled as sufficient and insufficient for sample size, respectively, according to whether the sample size for training met the minimum required sample size according to Riley et al all 3 criteria.

Supplementary Table S3 Factors associated with sufficient sample size according the strict requirement

Variable grouping	Univariable logistic analysis			Multivariable logistic analysis		
	OR	95% CI	P value	OR	95% CI	P value
Journal						
European Radiology	Ref		0.091	Ref		0.290
Insights into Imaging	0.555	0.199-1.548	0.261	0.525	0.149-1.852	0.317
Other	3.078	0.830-11.410	0.093	2.432	0.442-13.399	0.307
Study design						
Retrospective	Ref		0.399	n. a.		
Prospective	0.556	0.142-2.177	0.399			
Model testing method						
Development with only internal testing	Ref		<0.001	Ref		<0.001
Development only or with cross-validation	0.475	0.053-4.255	0.506	0.264	0.024-2.855	0.273
Development with only external testing	3.562	1.125-11.279	0.031	5.191	1.322-20.391	0.018
Development with internal and external testing	8.143	2.972-22.309	<0.001	15.812	4.429-56.453	<0.001
Imaging modality						
CT	Ref		0.170	n. a.		
MRI	0.451	0.197-1.036	0.061			
Other	0.781	0.226-2.699	0.696			
Specialty of topic						
Gastrointestinal	Ref		0.101	Ref		0.039
Neuro	1.667	0.427-6.499	0.462	1.814	0.376-8.750	0.458
Chest	4.333	1.098-17.107	0.036	8.335	1.546-44.924	0.014
Genitourinary	2.476	0.544-11.272	0.241	6.164	0.909-41.791	0.063
Breast	10.111	2.005-50.980	0.005	14.058	1.775-111.358	0.012
Cardiac and vascular	4.333	0.943-19.905	0.059	20.385	2.617-158.806	0.004
Other	2.667	0.764-9.312	0.124	4.282	0.927-19.785	0.063
Intend use						
Prognostic	Ref		0.577	n. a.		
Diagnostic	1.262	0.558-2.856	0.577			
Calibration metrics						
Absence	Ref		0.352	n. a.		
Presence	1.449	0.664-3.161	0.352			
Sample size justification						
Absence	Ref		0.193	n. a.		
Presence	2.300	0.657-8.055	0.193			
Sample size discussion						
Absence	Ref		0.381	n. a.		
Presence	0.691	0.302-1.579	0.381			
Reporting guideline						
None	Ref		0.146	n. a.		

Applied	2.252	0.753-6.733	0.146			

The study would be labeled as sufficient for sample size according to (1) a relatively weak requirement: if the sample size for training met the minimum required sample size according to Riley et al all 3 criteria; or (2) a relatively strict requirement: the total sample size and minimum required sample size according to Riley et al criterion 3. There are 42 and 74 studies are labeled as sufficient and insufficient for sample size, respectively, according to whether the total sample size met the minimum required sample size according to Riley et al criterion 3.

Supplementary Figure S1 Adherence to sample size requirements

Boxplots for the actual sample size (blue), the estimated minimum sample size (yellow), and the difference between them (red), considering the difference between the total sample size and minimum sample size according to Riley et al criterion 3 (left), and the sample size for training and minimum sample size according to Riley et al all 3 criteria (right), respectively.

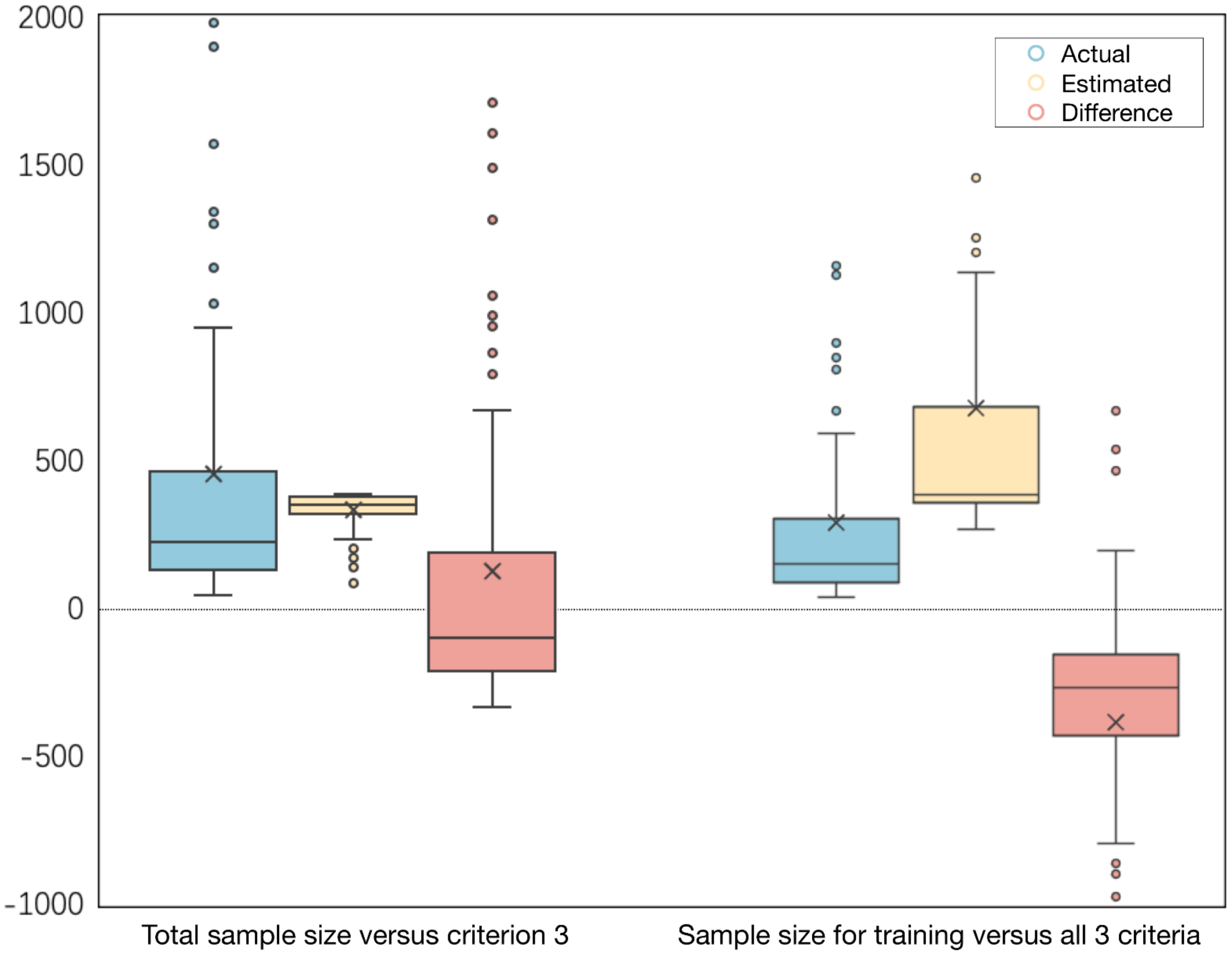

Supplement: Supplementary file 1 — ELECTRONIC SUPPLEMENTARY MATERIAL [file 330_2024_11331_MOESM1_ESM.pdf]
